# Supplementary material for: The Incidence of Scabies in Far North Queensland, Tropical Australia: Implications for Local Clinical Practice and Public Health Strategies
Source: Trop Med Infect Dis. 2025 Apr 18;10(4):111. doi: 10.3390/tropicalmed10040111 (PMC12031231; doi:10.3390/tropicalmed10040111)

Supplementary Table and Figures

Supplementary table 1. Summary of the 2020 International Alliance for the Control of Scabies Consensus Criteria for the Diagnosis of Scabies

**A. Confirmed scabies**
At least one of:
A1: Mites, eggs or faeces on light microscopy of skin samples
A2: Mites, eggs or faeces visualized on an individual using a high-powered imaging device
A3: Mite visualized on an individual using dermoscopy
**B. Clinical scabies**
At least one of:
B1: Scabies burrows
B2: Typical lesions affecting male genitalia
B3: Typical lesions in a typical distribution and two history features
**C. Suspected scabies**
One of:
C1: Typical lesions in a typical distribution and one history feature
C2: Atypical lesions or atypical distribution and two history features
History features
H1: Itch
H2: Positive contact history

Diagnosis can be made at one of the three levels (A, B or C). A diagnosis of clinical or suspected scabies should only be made if other differential diagnoses are considered less likely than scabies.

Supplementary figure 1: Flow diagram describing identification of skin scraping episodes for inclusion in the study

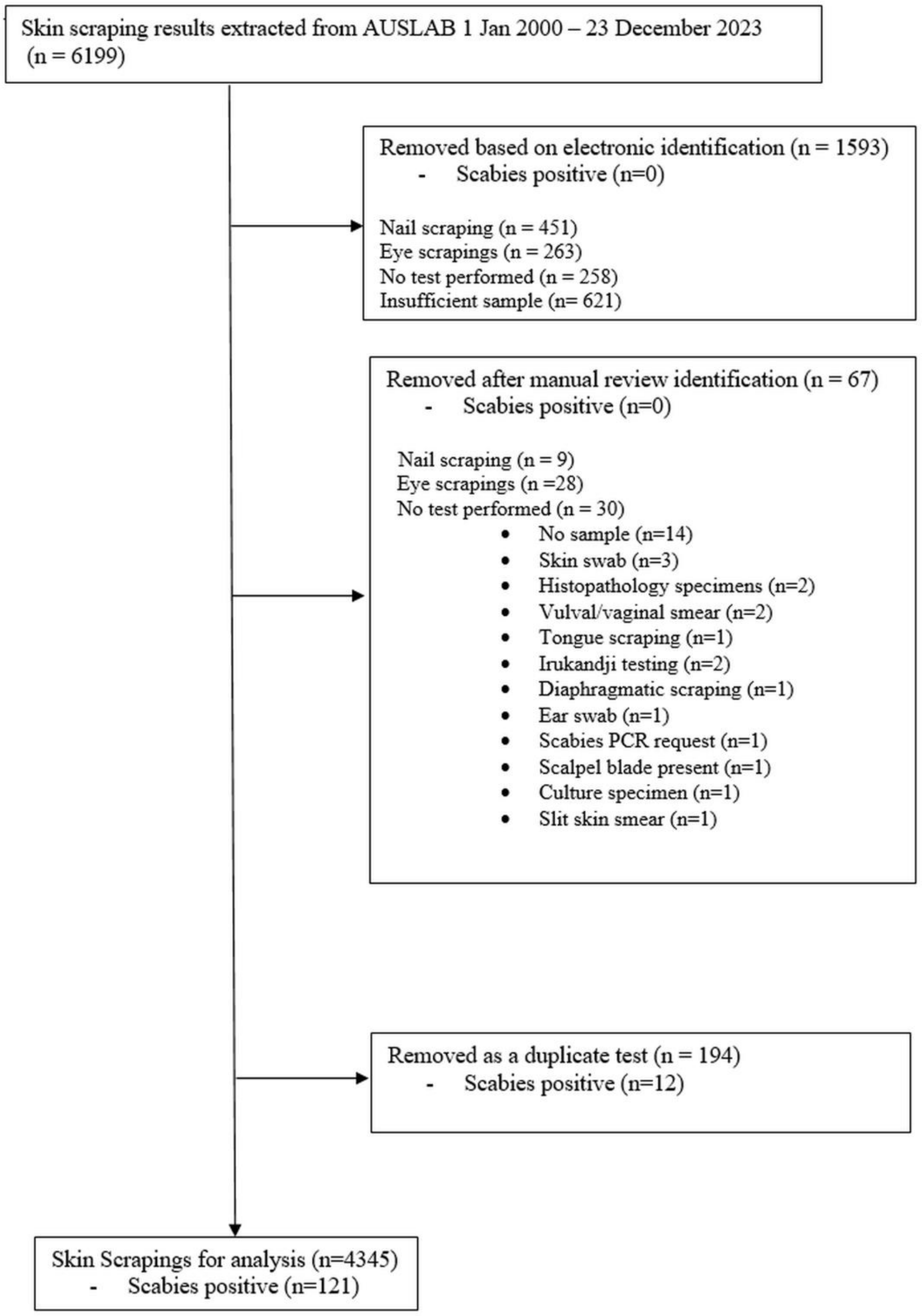

Supplementary figure 2: Flow diagram describing data management for the RHD analysis

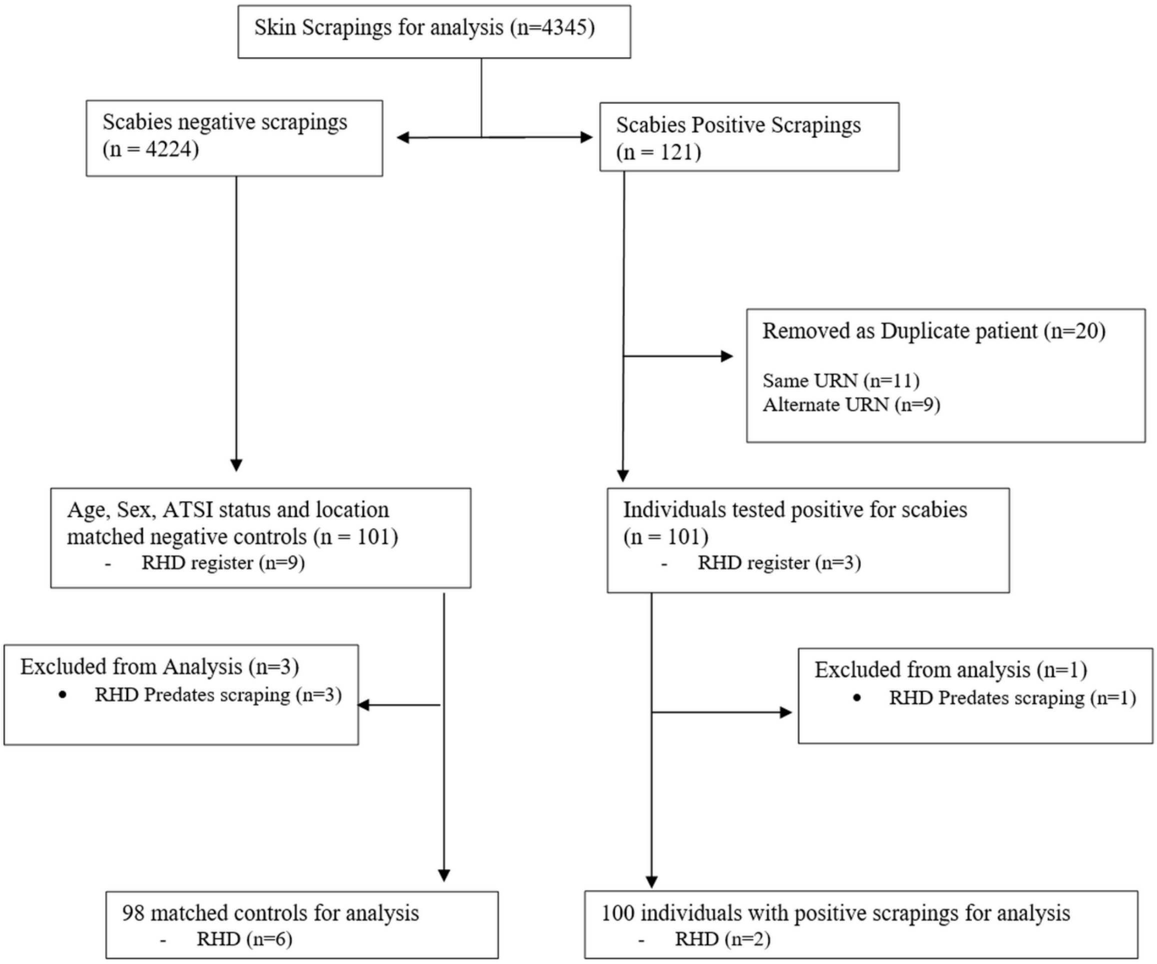

Supplementary figure 3: Flow diagram describing data management for the CKD analysis

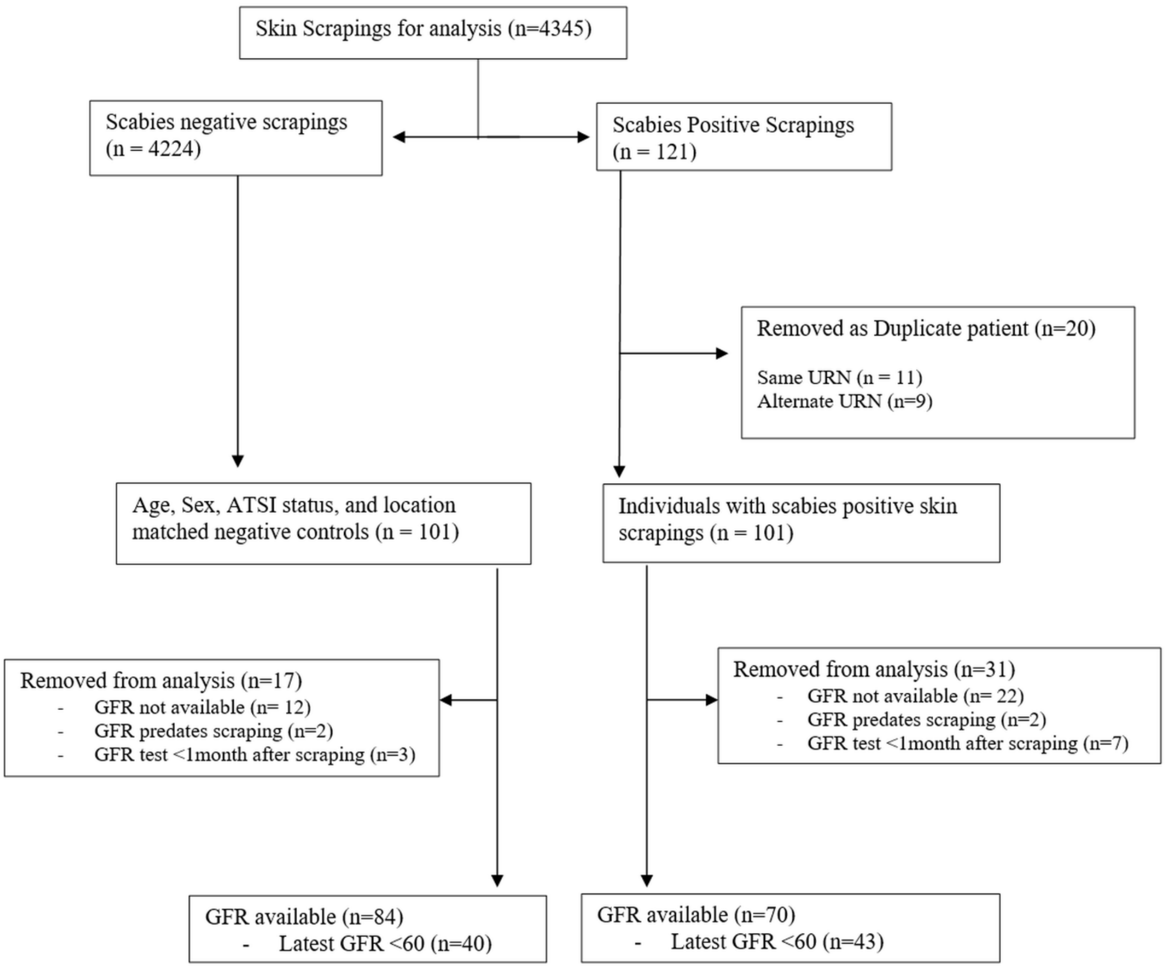

Supplementary figure 4: Flow diagram describing the presentations to Cairns Hospital Emergency Department

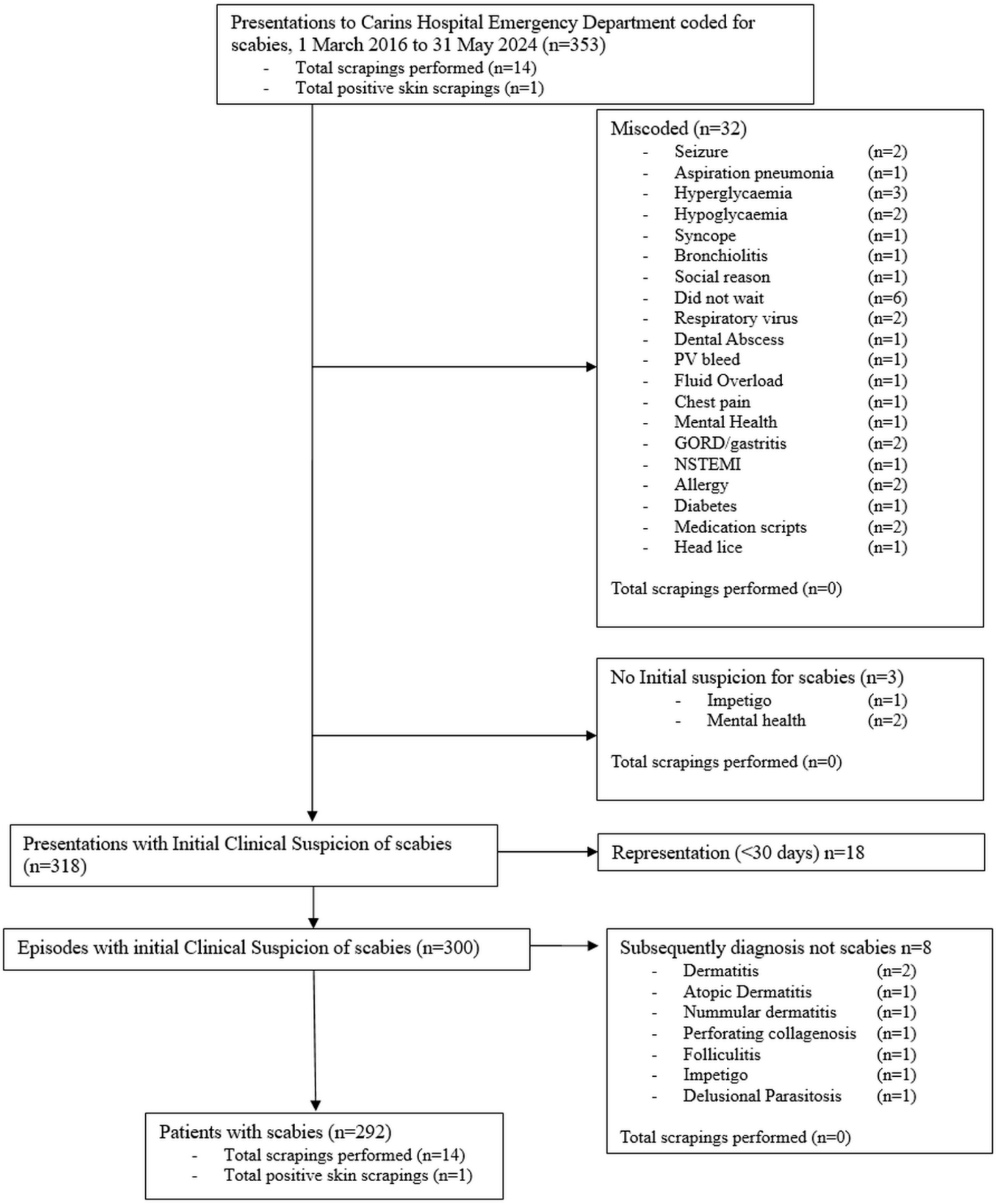

Supplement: Supplementary file 1 [file tropicalmed-10-00111-s001.zip › Revised supplementary table and figures.pdf]
